# A confocal Raman microscopic visualization of small penetrants in cellulose acetate using a deuterium-labeling technique

**DOI:** 10.1038/s41598-020-73464-8

**Published:** 2020-10-02

**Authors:** Hiroyuki Kubota, Koji Sakamoto, Toshiro Matsui

**Affiliations:** 1 Department of Bioscience and Biotechnology, Faculty of Agriculture, Graduate School of Kyushu University, 744 Motooka, Nishi-ku, Fukuoka, 819-0395 Japan; 2 Tobacco Science Research Center, Japan Tobacco Inc., 6-2 Umegaoka, Aoba-ku, Yokohama, Kanagawa 227-8512 Japan

**Keywords:** Chemistry, Analytical chemistry, Imaging studies

## Abstract

The purpose of the present study was to visualize the sorption dynamics of small compounds, such as propylene glycol (PG) in cellulose acetate (CA) film, by deuterium (d) labeling-aided confocal Raman spectroscopy (CRM). Substitution of hydrogen atoms in the target molecule with deuterium caused a marked shift of C-H bond-related Raman bands to low wavenumbers, while the number of deuterium did not affect the magnitude of the shift. Raman bands derived from the stretching vibration of C–H near 2900 cm^−1^ for PG and ethanol were shifted to approximately 2100 cm^−1^ for PG-d6 and ethanol-d5 in the silent region of the CA Raman spectrum. When PG-d6 was dissolved in glycerol triacetate (GTA), the observed Raman intensity ratio at 2123 cm^−1^ of PG-d6 against 1739 cm^−1^ of GTA (C=O bond-related) showed a linear relationship between the molar and intensity ratios, indicating that the observed Raman intensity can be used for quantitative assay of the target in polymer film. The depth profiling experiments by CRM revealed that the distribution (or sorption) of PG-d6 in CA film was successfully visualized as a function of Raman band at the characteristic 2123 cm^−1^ intensity ratio.

## Introduction

Plastic polymer materials are widely used for the packaging of foods, cosmetics, and pharmaceuticals, etc.… In contrast to their prevalence based on economic and protecting properties, the quality of the foods packaged in these materials may deteriorate from light oxidation and/or sorption of flavors. In particular, marked flavor losses due to sorption have been reported for citrus and apple juices packed with polyolefin films such as polyethylene and polypropylene^1–3^.

In a previous series of studies on sorption, the magnitude and behavior of sorption into film was determined by the cohesive energy (or the Solubility Parameter, SP) of and between flavor and film, according to the Arrhenius equation^4,5^. However, the prediction of sorption according to the SP theory was restricted to homogenous polymer films, and not heterogeneous ones such as laminated packages, since the theory is based on the constant diffusivity of flavor penetrant in homogeneous film polymer. Thus, no observations on the distributing (or migrating) behavior of flavors in the inside of films have been reported due to the lack of appropriate analytical evaluations.

To date, direct measurements of sorbed flavor molecules in the inside of films have not been established, without disruption of flavor distribution. A confocal laser scanning microscope technique (CLSM) was proposed to establish sorption visualization in films^6,7^. However, the CLSM technique was only applicable to fluorescence compounds. A matrix-assisted laser desorption/ionization imaging mass spectrometry (MALDI-IMS)^8^ method was also applied for sorption visualization, and the local distribution of penetrants in laminated films was successfully visualized. The non-destructive MALDI-IMS visualization, however, still has serious disadvantages due to limited targets with low volatility and high ionization efficiency in the mass spectrometry (MS) vacuum chamber.

In this study, by considering the importance and benefit of non-destructive visualization of volatile flavor compounds in film polymers for food quality evaluation, a confocal Raman spectroscopy (CRM) was applied to a sorption study for the first time, since the CRM technique requires no molecular properties, such as less volatility and fluorescence, for targeting volatile flavors. The high visualization potential of CRM has been proven in reports on polymer materials and biological samples^9–12^. The only limitation of target molecules for the CRM assay is that the targets have characteristic and specific Raman bands without any overlapping with bands derived from matrices. To date, several methods to solve the above-mentioned issue by alkyne tagging, diene tagging, or deuterium (D) labeling^13^ have been proposed. In this study, we applied the deuterium labeling technique for specific Raman shift of target volatiles to visualize the sorption dynamics inside of the polymer film. In this study, propylene glycol (PG) and ethanol, which are a solvent and humectant for foods, cosmetics, and pharmaceuticals were targeted for deuterium labeling penetrant as well as menthol as a model flavor. Cellulose acetate (CA) film was used as the target polymer, since we have already visually clarified by confocal laser scanning microscopy that a fluorescent penetrant can be sorbed into CA film in a Fick's second law-type manner^6,7^.

## Results

### Effect of deuterium labeling of targets on Raman spectra

**Figure 1:**
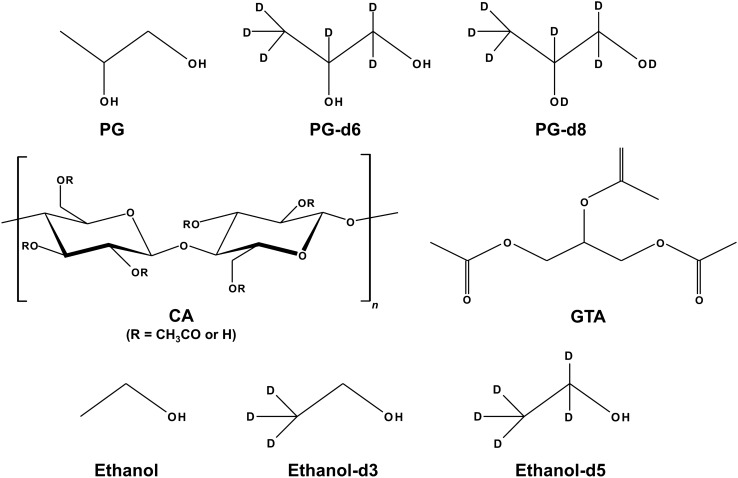
Chemical structures of compounds: PG, PG-d6, PG-d8, CA, GTA, ethanol, ethanol-d3, and ethanol-d5.

**Figure 2:**
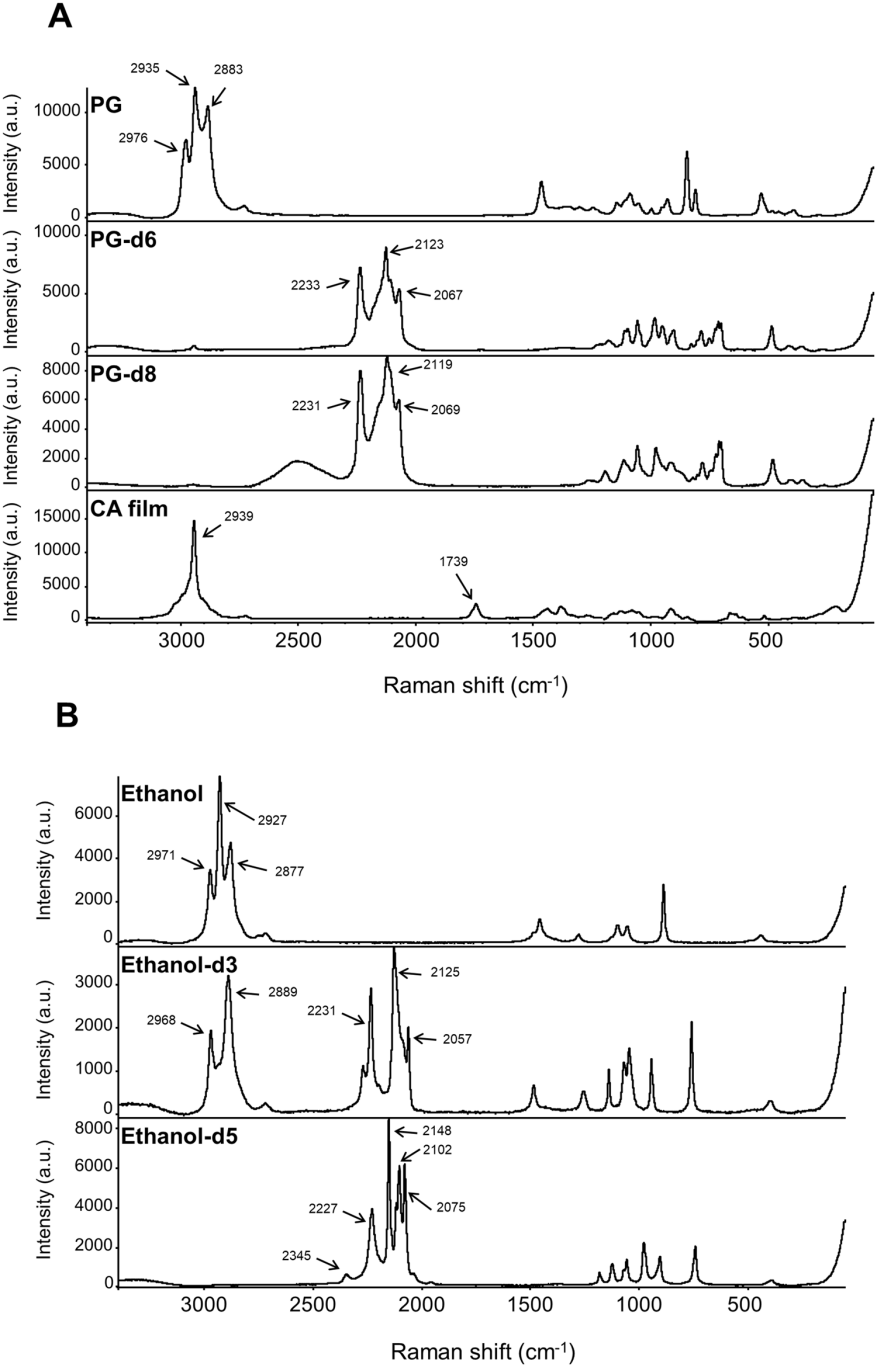
Raman spectra of compounds. (**A**) Raman spectra of PG, PG-d6, PG-d8, CA film, (**B**) ethanol, ethanol-d3, and ethanol-d5 acquired using CRM with an exposure time of 1 s at a laser excitation wavelength of 532 nm (power of 10 mW). Detailed analytical conditions are described in the Methods section.

Prior to the visualization of small penetrants absorbed inside polymer film by non-destructive CRM measurements, Raman bands from the targeting penetrant and polymer (Fig. 1) were characterized as to whether observed the bands were overlapped or not. As shown in Fig. 2A, the Raman spectra of PG were unfortunately overlapped with the targeting CA polymer film; in turn, no characteristic or specific Raman bands from the targets in this study were observed, and the proposed CRM technique could not apply for non-destructive visualization of the polymer-penetrant interaction. In contrast, it was found that the deuterium substitution of PG, namely PG-d6 and -d8, allowed a significant Raman shift from the C-H stretching vibration region of 2700–3100 cm^−1^^14^ to the silent region of 2000–2400 cm^−1^ for CA (Fig. 2A). Effective shifting to the silent region by deuterium labeling was also observed for ethanol^15^ (ethanol-d3 and -d5), as shown in Fig. 2B. Figure 2 also shows that the number of C-D bonds did not affect the magnitude of the shift of target Raman bands, indicating that the 2000–2400 cm^−1^ Raman band regions were characteristic or unique C–D stretching vibration regions that are not observed for natural C–H compounds. A specific band at around 2500 cm^−1^ in PG-d8 that is derived from O–D stretching vibration, which is shifted from O–H band at around 3400 cm^−1^^16^, would be excluded from the proposed CRM measurement due to its broadness. For the discriminant CRM visualization of CA polymer film from the targeting penetrants in this study (Fig. 1), a candidate Raman band would be 1739 cm^−1^ by stretching vibration of C=O ester bonds^17^ (Fig. 2A). This suggests that the sorption dynamics of small penetrants in polymer films may be evaluated by their own characteristic Raman bands.

### Quantitative Raman detection of deuterium labeled PG

**Figure 3:**
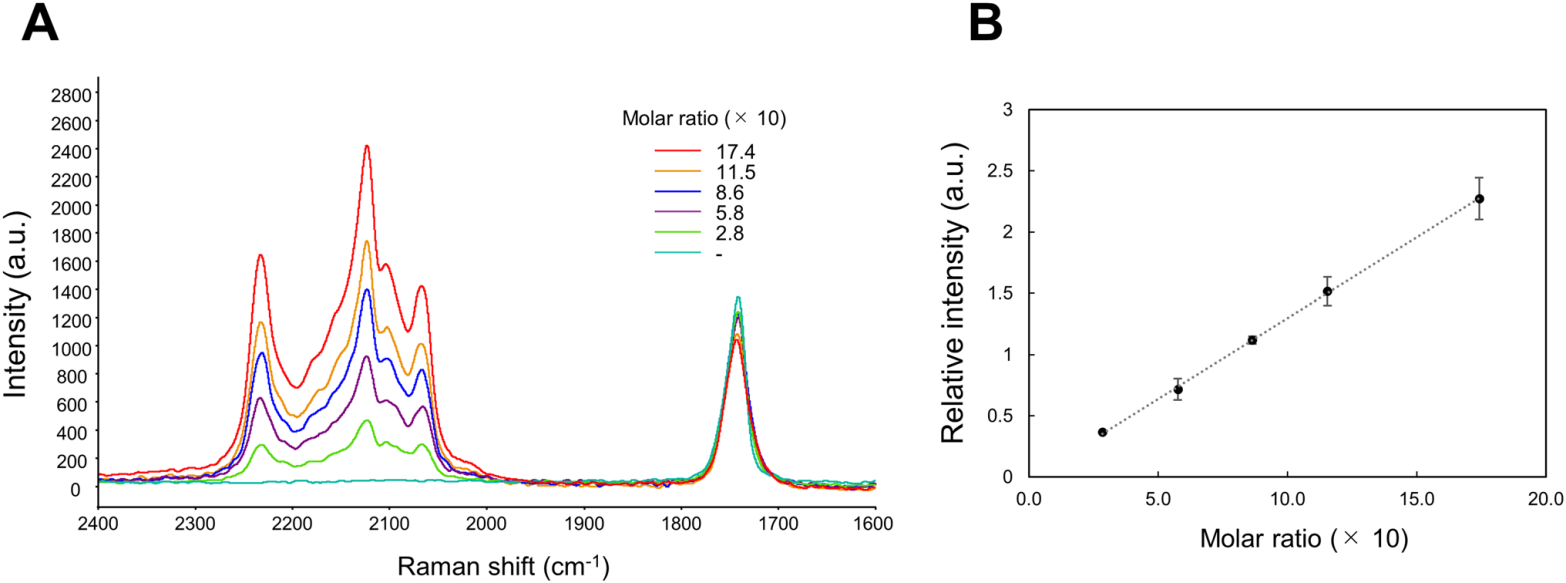
Relationship between the molar ratio of compounds and Raman intensity. (**A**) Raman spectra of different molar ratios of PG-d6 in GTA acquired using CRM with an exposure time of 1 s at a laser excitation wavelength of 532 nm (power of 10 mW). (**B**) Relationship between the molar ratio of compounds and relative Raman intensity. The relative Raman intensity was calculated using the band height ratio of PG-d6 characteristic band (2123 cm^−1^) to GTA characteristic band (1739 cm^−1^). The values represent the means ± S.D. n = 3. Detailed analytical conditions are described in the Methods section.

In order to confirm whether the CRM measurement could be used for the quantitative sorption evaluation assay, the concentration dependency of Raman band intensity of PG-d6 was examined by monitoring the characteristic C–D bond-derived Raman bands. In this study, glycerol triacetate (GTA) was used as the solvent for PG-d6, since it possesses C=O ester bond showing 1739 cm^−1^ stretching vibration^18^ (Fig. 3A), the same Raman band as the targeting CA polymer film (Fig. 2A). As shown in Fig. 3A, the observed C–D Raman band intensities (2000–2400 cm^−1^) increased with increasing PG-d6 concentration (molar ratio of PG-d6 to GTA, 0.28 to 1.74). It was clear that the intensity ratio of the novel Raman band (2123 cm^−1^) from PG-d6 to the intensity of 1739 cm^−1^ from GTA gave a good linearity with PG-d6 concentration (correlation coefficient of 0.999). This suggests that quantitative sorption analysis of a small penetrant (in this study, PG-d6) in polymer film can be achieved by using the proposed CRM visualization technique.

### Time-course depth profiling on the penetration process of PG-d6 into CA film

**Figure 4:**
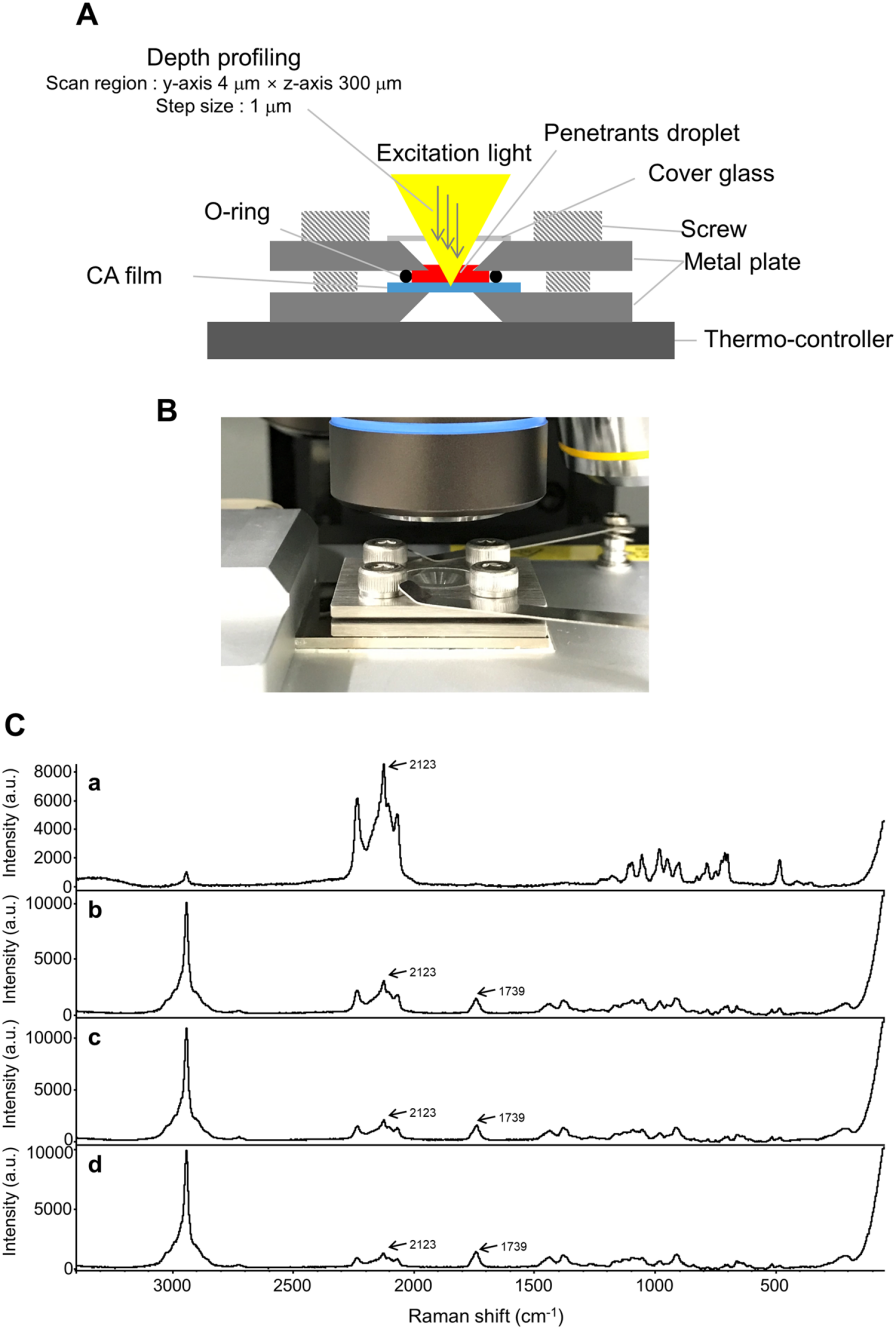
Images of the experimental set-up for depth profiling of PG-d6 into CA film and Raman spectra collected at different scanning depths during sorption process. CA film were sandwiched in the vertical direction by metal plates with a center hole. The film and the penetrant thereon were depth scanned through the hole with CRM in the range of 4 × 300 µm (y-, z-axis) with 1 µm steps (**A**: cross sectional image, **B**: graphical image). (**C**) Raman spectra collected at different scanning depths from the penetrant to the bottom of the film. Raman spectra above the CA film (a) and 20 (b), 50 (c), and 80 µm (d) from the film surface were acquired using CRM at 168 h after PG-d6 adding by droplet onto the film. Raman spectra were recorded with an exposure time of 1 s at a laser excitation wavelength of 532 nm (power of 10 mW). Detailed analytical conditions are described in the Methods section.

Depth profiling of the sorption process of PG-d6 into CA film was performed by the proposed CRM analysis according to the experimental setup shown in Fig. 4A,B. This film fixture enabled reproducible measurements for various types of polymer films and penetrants without leakage or volatilization of the penetrants during lengthy measurements. Raman spectra above CA film and 20, 50, and 80 µm from the film surface in scanning depth were acquired using CRM at 168 h after adding PG-d6 by droplet onto the film (Fig. 4C). Characteristic and intense bands of PG-d6 were observed at 2123 cm^−1^ even in CA film and the intensity became weaker closer to the bottom of the film. This indicated that the time-course distribution of PG-d6 in CA film could be quantitatively evaluated using the non-destructive CRM measurement.

**Figure 5:**
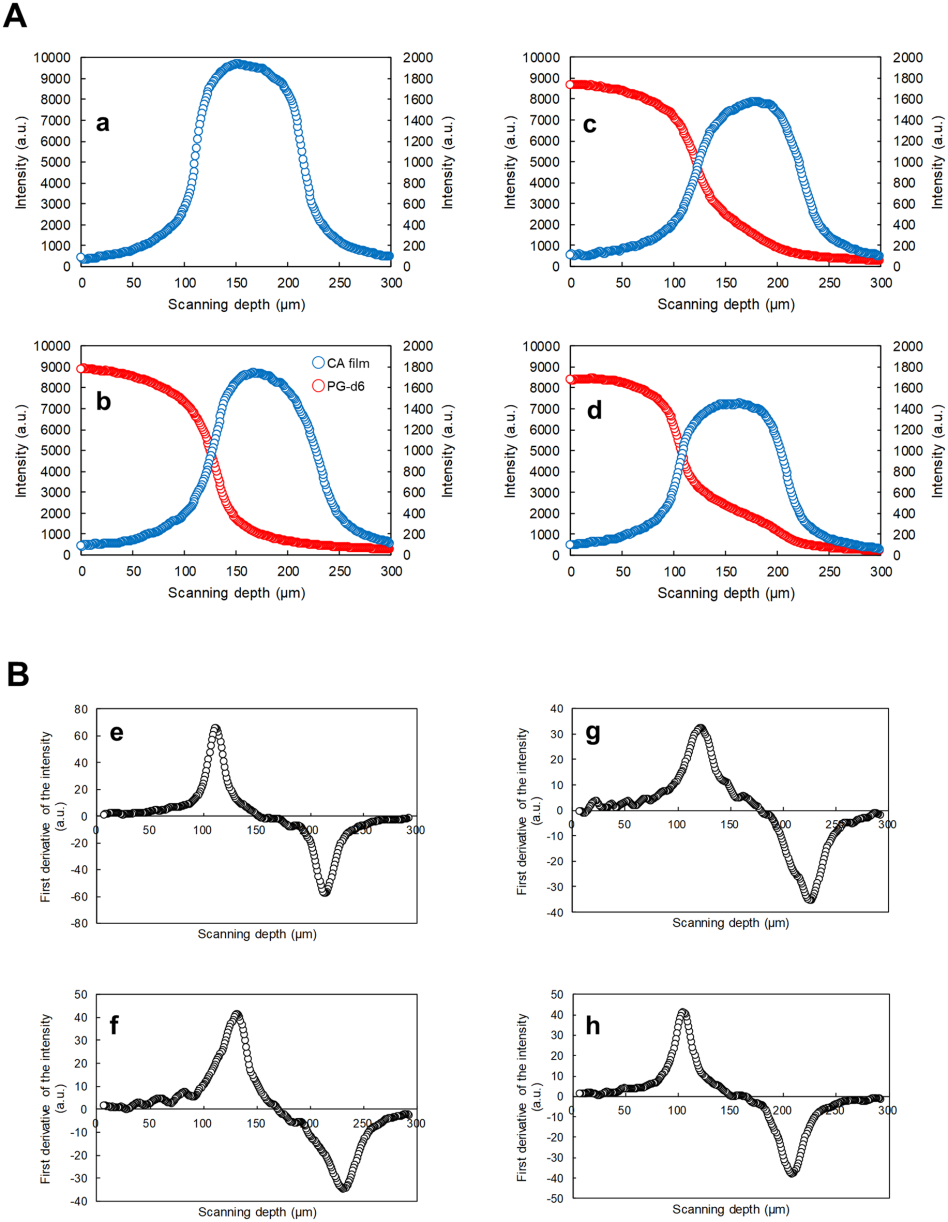
Time-course depth profile on penetration process of PG-d6 into CA film. (**A**) The intensity curves toward to the depth direction before the addition of PG-d6 onto CA film (a) and 5 min (b), 96 h (c) and 336 h (d) after the PG-d6 addition are shown with the band height at 2123 cm^−1^ for PG-d6 (open red circles) and 1739 cm^−1^ for CA film (open blue circles). (**B**) The first derivative of each intensity curve before PG-d6 addition (e), and 5 min (f), 96 h (g) and 336 h (h) after the addition of PG-d6 is shown. The scanning depth is shown in nominal focus position. Detailed analytical conditions are described in the Methods section.

**Figure 6:**
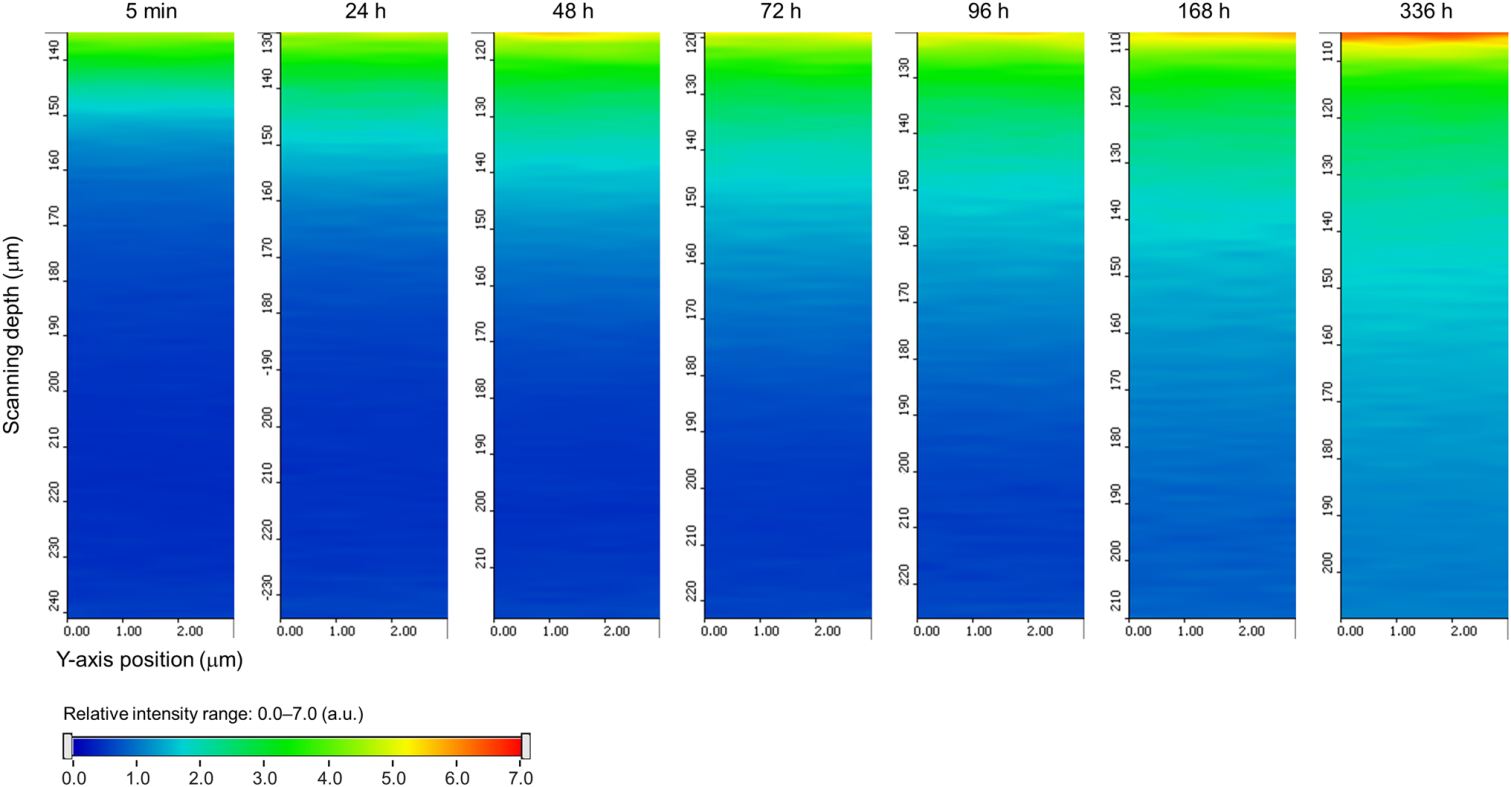
Time-course chemical images of relative Raman intensity of PG-d6 to CA in the film area. The rainbow color in the range of 0.0–7.0 shows the relative intensity calculated with the band to height ratio of PG-d6 characteristic band (2123 cm^−1^) to CA characteristic band (1739 cm^−1^) in CA film region. The depth ranges were cut out with positions of maximum and minimum values in the first derivative Raman intensity curve with the CA characteristic band. The scanning depth is described in the nominal focus position. Detailed analytical conditions are described in the Methods section.

Figure 5A provides the time-course intensity curves towards the depth direction with the specific bands of PG-d6 and CA film. In the measurement, the Raman intensity of PG-d6 existing inside the CA film increased over time, and the process of PG-d6 penetration into CA film was clearly observed. The first derivative of each intensity curve is shown in Fig. 5B. The depth range of the film can be deduced from the maximum and minimum values of the bands in the first derivative of the intensity curves^19^. The depth range of the film was calculated from the first derivative intensity curve before adding the penetrants, which was 99.7 ± 2.52 µm in nominal focus position. The actual film thickness was calculated to be 149 ± 3.75 µm in consideration of the refractive index ratio of CA film (*n* = 1.49) to that of air (~ 1). This corresponded to the actual measured value of the film thickness (150 ± 1.14 µm), thus the calculation of the film depth range with the first derivative was appropriate. Figure 6 shows the y–z axis chemical images of the relative Raman intensity of PG-d6 to CA film in the calculated depth range. The relative intensity, with a specific band height ratio of PG-d6 to CA film in the film y–z axis area, was shown as rainbow color in the range of 0.0–7.0. It is clear from the figure, the PG-d6 amount in the film increased with time in the depth direction, and the sorption process of small molecules in the polymer could be visually observed with the deuterium labeling-aided CRM. In addition, even if relatively weak bands, 2067 cm^−1^ and 2233 cm^−1^, rather than 2123 cm^−1^, were used as indicator bands of PG-d6, there was no significant difference for visualizing the penetration process (Supplementary Figs. S1 and S2).

### Discriminant detection of a mixture of penetrants in depth profiling

**Figure 7:**
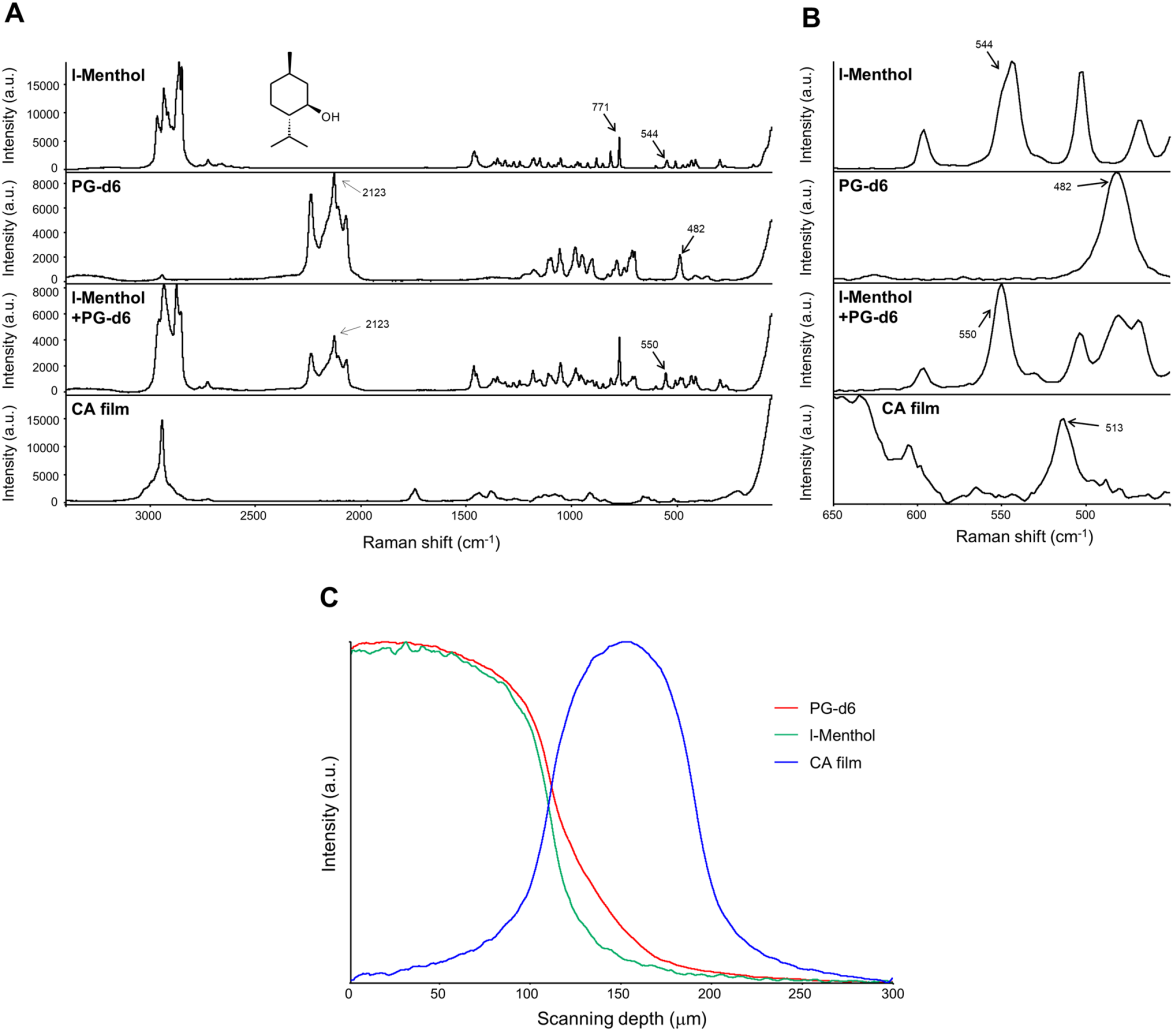
Raman spectra and depth profile of compounds. (**A**) Raman spectra of l-menthol, PG-d6, l-menthol/PG-d6 solution, and CA film acquired using CRM with an exposure time of 1 s at a laser excitation wavelength of 532 nm (power of 10 mW). (**B**) Enlarged view in wavenumber range of 450–650 cm^−1^ in full scale. (**C**) Depth profile on penetration process of l-menthol and PG-d6 into CA film at 144 h after adding penetrants onto film. The intensity curves toward to the depth direction are shown with the band height at 2123 cm^−1^ for PG-d6 (red line), 550 cm^−1^ for 1-menthol (green line), and 1739 cm^−1^ for CA film (blue line). The scanning depth is shown in the nominal focus position. Detailed analytical conditions are described in the Methods section.

A mixture of penetrants was measured by the proposed deuterium-labeling CRM, in which l-menthol, as a typical flavor, and PG were used (Fig. 7). Although in the case of a mixture of l-menthol and PG without deuterium, an isolated Raman band at 771 cm^−1^ from breathing vibration of cyclohexane ring of l-menthol^20,21^ was detected, and isolated bands from PG were not detected (Supplementary Fig. S3). In contrast, as shown in Fig. 7A,B, a mixture of l-menthol and PG-d6 could be differentiated by their isolated Raman bands at 550 cm^−1^ (assigned to cyclohexane ring deformation^22^) and 2123 cm^−1^, respectively, without any overlap with CA film matrices. At 144 h after adding by droplet a mixture of l-menthol and PG-d6 solution onto CA film, l-menthol and PG-d6 with their Raman bands were detected inside the CA film, successfully providing individual depth profiling curves in CA film (Fig. 7C). Taken together, the band shift by deuterium labeling not only enables identification of a single penetrant, but also it improves the molecular discrimination, even for plurality of penetrants. Consequently, the deuterium labeling-aided CRM would be highly applicable for visualization analysis of sorption dynamics of flavor penetrants in polymer films.

## Discussion

Raman spectroscopy is a method of detecting Raman scattering light with a wavelength different from that of incident light due to natural vibration of bonds in molecules. Adding the confocal system to Raman spectrometry enables three-dimensional spectroscopic analysis. Therefore, the CRM is widely used as a means of local analysis of polymers^9,10,23,24^. In addition, the CRM does not require vacuum condition, so that it is suitable for measuring the penetration process of volatile small molecules such as flavors into polymers.

As shown in Fig. 2A, characteristic and intense bands that do not overlap with bands from CA film could not be found in the PG spectrum. In this case, the solution could be generating other Raman bands in the spectrum of the penetrants. The previous study^13^ showed that characteristic band(s) could be generated in the silent region of live cells of Raman spectrum (1800–2800 cm^−1^) with alkynyl, nitrile, azide group introduction to target molecules or with deuterium substitution of hydrogen in the molecule. The CA film employed in the present study also has a silent region (1800–2700 cm^−1^) (Fig. 2A), therefore it is reasonable to introduce the tags to PG. Among the candidates, deuterium labeling is appropriate to minimize the impact on sorption dynamics, since the target molecules used in this study were small and volatile.

Each band appearing in the Raman spectrum corresponds to a molecular vibration. Assuming that each vibration mode is a harmonic frequency, the wavenumber of natural frequency in the harmonic oscillator is expressed by the following equation:^25^

1 $$\stackrel{-}{{\varvec{\nu}}}=\frac{1}{2{\varvec{\pi}}{\varvec{c}}}\sqrt{\frac{{\varvec{k}}}{{\varvec{\mu}}}}$$

where *µ* is the reduced mass, that is the harmonic mean of the masses *m*_*1*_ and *m*_*2*_, of atoms moving in vibration motion, *k* is the force constant determined by the strength of the bond between the atoms relating to amplitude of the atoms motion and *c* is the velocity of light. When C–H bonds were replaced with C–D bonds, Raman bands shifted from around 2900 cm^−1^ to around 2100 cm^−1^ (Fig. 2). This can be explained to be a result of the increase in reduced mass. Even with the same C–D bonds, multiple bands appeared because they reflect the various environments of molecules. Referring to the Raman spectra of ethanol in Fig. 2B, the maximum intensity of C–D stretching vibration increased as the number of deuterium substitutions increased. This is thought to be due to the increase of C–D bonds at the focus of the confocal laser. Meanwhile, PG also has O–H bonds. Despite the fact that the alcohol-type O–H stretching vibration appears in 3200–3600 cm^−1^, these Raman intensities are generally weak and broad^15,16^. In fact, PG-d8 in which O–H bonds were replaced with O-D bonds was measured by CRM, however, a weak band around 2500 cm^−1^ appeared to be broad (Fig. 2A). This result suggests that the band which was derived from the O–D bond is inappropriate as an indicator band of the penetrant. From the above, deuterium labeling is effective for visualizing the sorption dynamics of small molecules, and a higher deuterium substitution degree can result in a higher intensity. Further, substitution of deuterium into hydroxyl groups is not effective.

In this study, the penetrating process of PG-d6 into CA film with time was clearly visualized (Fig. 6). Furthermore, distributed multiple components (l-menthol and PG-d6) in these penetration processes were simultaneously detected (Fig. 7). In previous studies^26–28^, the methods of measuring the concentration of absorbed penetrants in the bulk were the mainstream. In contrast, this would be the first-of-its-kind of in situ visualization of volatile small molecules distribution during sorption into the polymer.

Raman scattering generally has 10^−8^ smaller cross-sections than other spectroscopic processes such as infrared spectroscopy (IR)^29^, so that the detection limit of additives in polymers or components in solutions of spontaneous Raman spectroscopy is on the order of several percent by weight. The concentration range in the concentration-dependency evaluation (Fig. 3) was 4.18–39.6 wt%. For instance, in the case of not only PG but also GTA, which is a general plasticizer for CA, the respective spectra of GTA and CA overlapped with each other, causing no characteristic Raman band detection (Supplementary Fig. S4). However, deuterium labeling enabled detection at the same level as systems of general additives and polymers. Indeed, further work is necessary to increase the sensitivity for applications to trace flavor analysis. For this, it is conceivable to consolidate this technique with a highly sensitive technique such as surface-enhanced Raman scattering (SERS)^29^ using metal nanoparticles for the cross-sectional imaging of a penetrated film. As for the sorption of small molecules into polymers, a Fickian model has been reported for the evaluation of diffusion coefficient^7,19,23,24^. The relative Raman intensity obtained by the present proposed Raman assay may be applied for semi-quantitative evaluation of penetrated compounds; a study is currently ongoing to confirm the quantitative assay of the deuterium-aided Raman spectroscopy by fitting the results (Fig. 6) with a Fickian model. In the future, it would be possible to analyze local sorption dynamics based on the physicochemical property of penetrants by comparing diffusion coefficients in an extension of this method. Deuterium labeling with Raman spectroscopy is used in the field of biochemistry^30,31^. The present study demonstrated that this deuterium labeling-aided CRM technique is also useful for understanding the sorption behavior of small molecules, even in polymers.

To conclude, in this study, we visualized the penetration process of PG-d6 in CA film by applying the deuterium labeling-aided CRM with non-fluorescent labeling and non-destructive under atmospheric pressure conditions. Raman band shifts from deuterium labeling could markedly improve the differentiation of the small molecules compared to the polymer. This technique can be applied to systems consisting of other volatile compounds and polymers which do not have characteristic Raman bands, such as alkynyl and nitrile groups. Based on this study, it would be possible to elucidate the sorption process of targeted flavors (e.g., limonene, the key flavor of citrus^1,2^) in matrices, and that of monomers or plasticizers present in polymers by the effective use of deuterium labeling. As for future research prospects, expansion of this technique to sorption kinetics studies for flavors in micro areas such as multi-layered films and microfibers is expected.

## Methods

### Materials

PG (Mw, 76.10 g/mol), GTA (Mw, 218.2 g/mol), ethanol (Mw, 46.07 g/mol), and l-menthol (Mw, 156.27 g/mol) were purchased from Fujifilm Wako Pure Chemical Co. (Osaka, Japan). PG-d6 (Mw, 82.13 g/mol), PG-d8 (Mw, 84.14 g/mol), ethanol-d3 (Mw, 49.09 g/mol), ethanol-d5 (Mw, 51.10 g/mol) were obtained from Cambridge Isotope Laboratories Inc. (Tewksbury, MA, USA). All these chemicals were used without further purification. CA film [refractive index: *n* = 1.49, acetyl value = 55.5 ± 0.4, plasticizer, mainly glycerol triacetate: 19% (w/w)] of 150 µm thickness with flat surface were provided by Celanese Co. (TX, US) and used without further treatment.

### CRM measurements

CRM experiments were performed on a Thermo Fisher Scientific DXR2xi system equipped with a diode-pumped solid-state laser (532 nm excitation). The power of the laser was 10.0 mW. The laser beam was focused on the sample through a dry objective lens (Olympus, LMPlanFL N, 50×, NA = 0.5). A 25 µm pinhole was used for the confocal aperture. The DXR2xi system used is also equipped with an electron multiplied charge-coupled device (EM CCD) and EM gain parameter. The parameter controlling the electron multiplying gain of the detector camera increases the signal to overcome the readout noise, which also increases other sources of noise. In this study, all measurements were performed with EM gain turned off and with a longer exposure time in order to obtain each spectrum with high S/N. The DXR2xi system was operated with the Thermo Scientific OMNICxi software and the acquired spectra were processed using the Thermo Scientific OMNIC software. Data acquisition consisted of 1 s exposure time. The number of exposures for the average of the spectrum was 2 for point analysis or 1 for depth profiling. A baseline correction process using polynomial approximation on OMNICxi was applied for all recorded spectra, in order to improve band discrimination of individual spectra by preventing undesirable disturbances or artifacts, such as fluorescence. Optical alignment and wave number calibration of the spectrometer was carried out with white light and polystyrene before measurement sessions.

### CRM point analysis of materials

PG, PG-d6, GTA, CA film, l-menthol, l-menthol + PG solution, and l-menthol + PG-d6 solution were placed on the slide glass, and ethanol, ethanol-d3, ethanol-d5 were poured in the transparent glass vial to obtain Raman spectra. For the concentration dependence experiment of the penetrant on CRM measurement, PG-d6/GTA solutions were prepared at room temperature by the dissolution of PG-d6 in GTA to reach molar ratio ranging from 0.28 to 1.74. The molar ratio of PG-d6 to GTA was defined as the molar ratio of PG-d6 to GTA. The mixtures were placed in a transparent glass vial and measured Raman spectra through the glass. Smoothing treatment was applied to the recorded spectra for concentration-dependency evaluation. Three replicates of Raman analysis for each solution (±the standard deviation) were performed for this experiment. For simultaneous detection of multiple penetrants in CA film, l-menthol/PG and l-menthol/PG-d6 solution were prepared in the above-mentioned manner to reach a molar ratio of 0.49 (l-menthol to PGs).

### Time-course depth profiling on penetration process of penetrants into CA film

A cross sectional image and a graphic image of the experimental setup for depth profiling are shown in Fig. 4A,B. The CA film with O-ring were sandwiched in the vertical direction by stainless steel plates (W30 × D30 × H2 mm) which have a 45° taper around a φ 4.0 center hole. CA film was set in the fixture and then applied to measurements after adding by droplet 10 µL of PG-d6 or l-menthol/PG-d6 solution onto the film. The film and penetrants thereon were depth scanned through the hole with CRM in the range of 4 × 300 µm (y-, z-axis) with 1 µm steps. In this experiment, specific penetrants were added by droplet onto the top of the polymer film, and measurement and storage were repeated in turn during the experiment to obtain depth profiles. Therefore, in this case, an immersion lens is not suitable, although the lens is usually applied to obtain high spatial resolution by filling a space between an object and the lens with a specific liquid such as water or oil. When measuring the inside of the object using a dry lens, the distance that the laser focus is physically moved (nominal focus position; NFP) is different from the actual distance that the laser focus has moved (actual focus position; AFP) due to the difference in the refractive index. The following formula has been reported for calibration of the laser focus moving distance:^32,33^.

2 $${A}{F}{P} = \frac{{tan}{{\alpha }}_{1}}{{tan}{{\alpha }}_{2}} {N}{F}{P}= \frac{{tan}\left[{{s}{i}{n}}^{-1}\left(\frac{{N}{A}}{{{\varvec{n}}}_{1}}\right)\right]}{{tan}\left[{{s}{i}{n}}^{-1}\left(\frac{{N}{A}}{{{\varvec{n}}}_{2}}\right)\right]} {N}{F}{P}$$

where *α*_1_ is the aperture angle and therefore the angle of incidence of the marginal rays and *α*_2_ is the corresponding refracted angle, *n*_l_ and *n*_2_ are the indices of refraction in the first and the second mediums, respectively and NA is the numerical aperture of the microscope objective. For low numerical apertures (e.g. below 0.65), Eq. (2) simplifies to:^32,34^

3 $${A}{F}{P}{ }=\frac{{{\varvec{n}}}_{2}}{{{\varvec{n}}}_{1}} {N}{F}{P}$$

and it was used for correcting mismatches in this study.

The depth profiles were measured by acquiring Raman spectra at different depths by moving the sample stage. Although the film and the penetrant were exposed to the laser beam, no deformation of the film could be detected by optical microscope observation and spectra reproducibility was demonstrated, thus possible damage of the materials seems to have been avoided. The Raman measurement was performed at room temperature (22 °C) on the thermo-controlling stage (PE120, Linkam Scientific Instruments Ltd., Tadworth, Surrey, UK), and storage was carried out in a desiccator with fresh silica gel at room temperature (22 °C). The obtained four lines of spectral data in the depth direction were averaged, smoothed, and then described as the band height intensity curve in the z-axis (scanning depth) direction. In depth profiling, the position of the film is deduced from the maximum and minimum values ​​of the first derivative of the Raman intensity curve in the depth direction^19^. Therefore, the band height intensity curve in the z-axis direction was differentiated using first order (Norris algorithm), and then CA film thickness and z-axis coordinates were calculated. Three replicates of time-course depth profiling were performed for this experiment.

## Supplementary information

Supplementary information

## Data Availability

The data supporting the findings reported herein are available from the corresponding author on request.
