# Supplementary material for: A confocal Raman microscopic visualization of small penetrants in cellulose acetate using a deuterium-labeling technique
Source: Sci Rep. 2020 Oct 2;10:16426. doi: 10.1038/s41598-020-73464-8 (PMC7532217; doi:10.1038/s41598-020-73464-8)
Supplement: Supplementary file 1 — Supplementary information [file 41598_2020_73464_MOESM1_ESM.pdf]

**Supplementary Information**

# A confocal Raman microscopic visualization of small penetrants in cellulose acetate using a deuterium-labeling technique

**Hiroyuki Kubota**[1,2*], **Koji Sakamoto**[2] **and Toshiro Matsui**[1]

[1] Department of Bioscience and Biotechnology, Faculty of Agriculture, Graduate School of Kyushu University, 744 Motooka, Nishi-ku, Fukuoka, 819-0395, Japan.
[2] Tobacco Science Research Center, Japan Tobacco Inc., 6-2 Umegaoka, Aoba-ku, Yokohama, Kanagawa, 227-8512, Japan

*Correspondence to: Hiroyuki Kubota, Tobacco Science Research Center, Japan Tobacco Inc., 6-2 Umegaoka, Aoba-ku, Yokohama, Kanagawa, 227-8512, Japan
E-mail: hiroyuki.kubota@jt.com
FAX: +81-45-973-6781

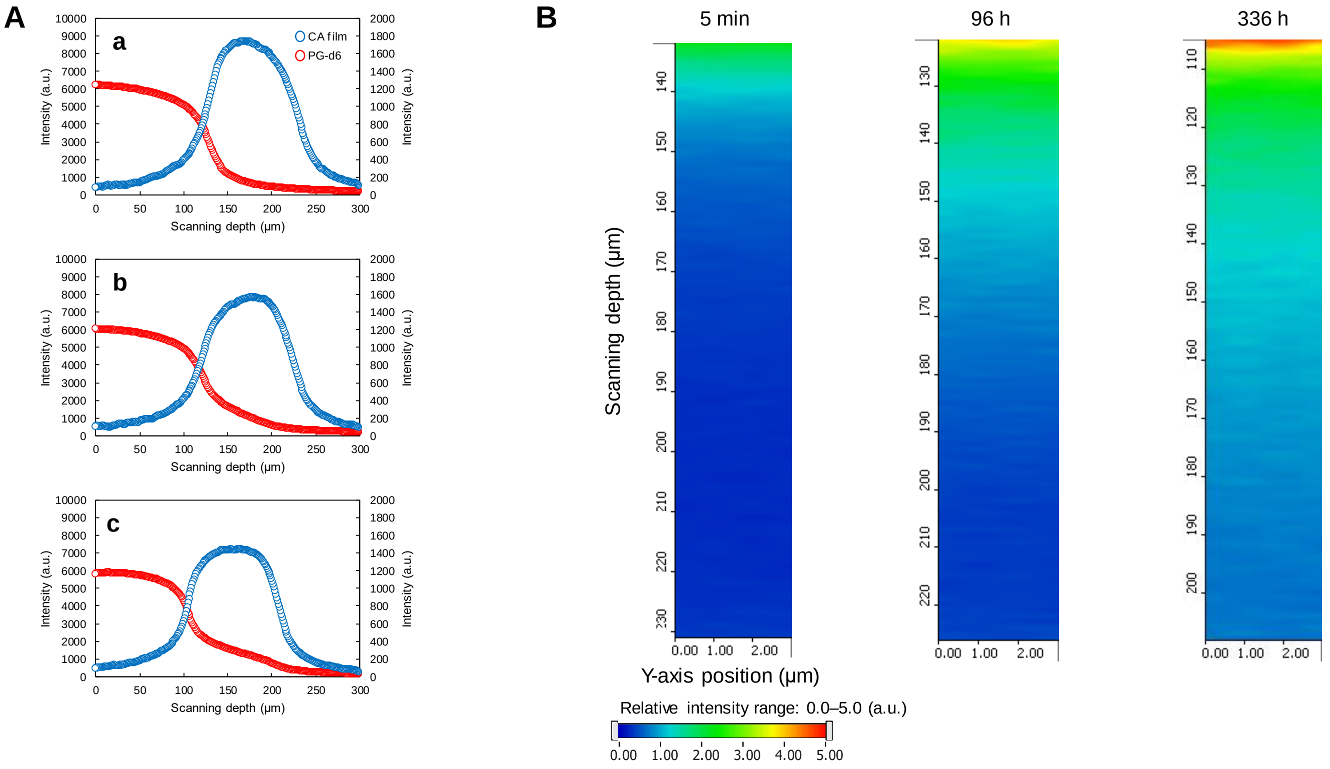


Supplementary Figure S1. Time-course depth profiling and chemical images of penetration process of PG-d6 into CA film. (A) The intensity curves towards the depth direction at 5 minutes (a), 96 hours (b), and 336 hours (c) after adding by droplet PG-d6 onto the film are shown with the band height at 2233 $cm^{-1}$ for PG-d6 (open red circles) and at 1739 $cm^{-1}$ for CA film (open blue circles). (B) Time-course chemical images of relative Raman intensity of PG-d6 to CA in the film area. The rainbow color in the range of 0.0–5.0 shows the relative intensity calculated with band height ratio of PG-d6 characteristic band to CA characteristic band in CA film region. The depth ranges were cut out with positions of maximum and minimum values in the first derivative of the Raman intensity curve with the CA characteristic band. The scanning film depth is described in nominal focus position. Detailed analytical conditions are described in the Methods section.

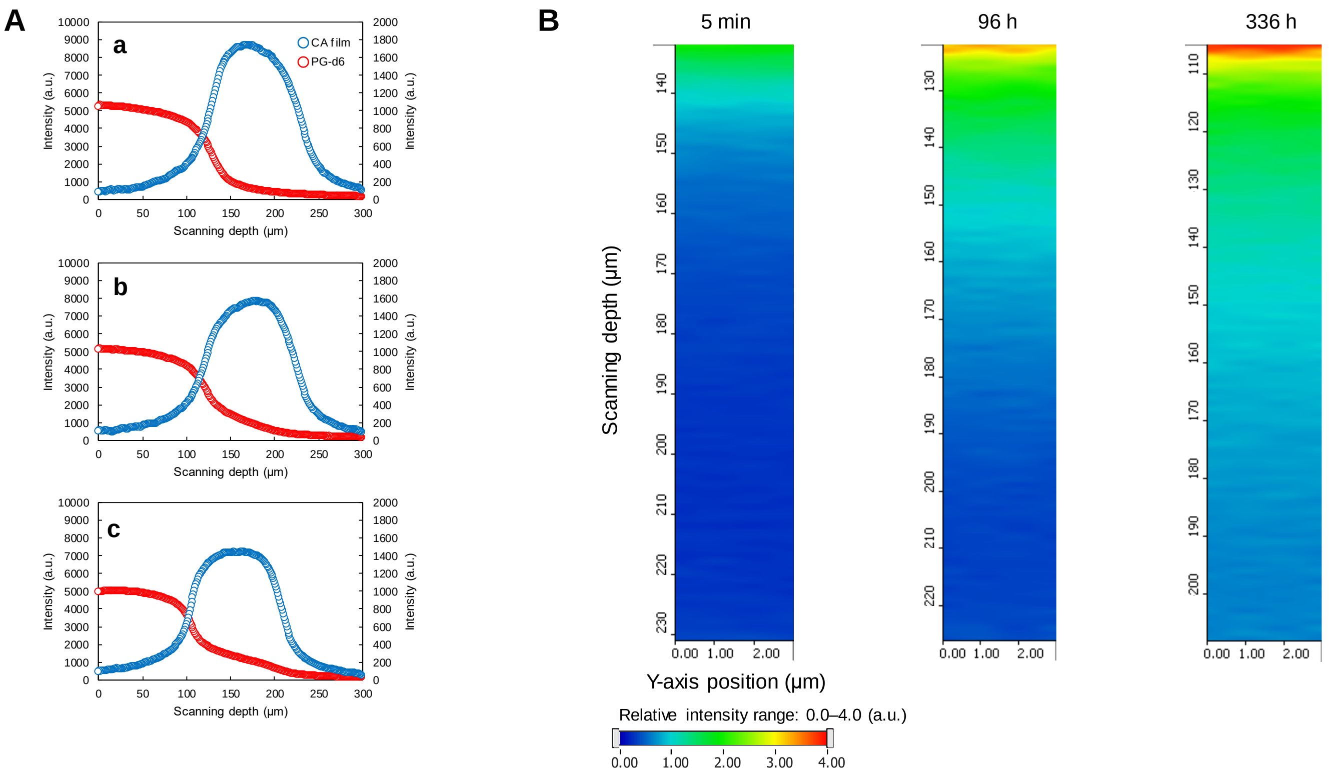


Supplementary Figure S2. Time-course depth profiling and chemical images of penetration process of PG-d6 into CA film. (A) The intensity curves towards the depth direction at 5 minutes (a), 96 hours (b), and 336 hours (c) after adding by droplet PG-d6 onto the film are shown with the band height at 2067 $cm^{-1}$ for PG-d6 (open red circles) and at 1739 $cm^{-1}$ for CA film (open blue circles). (B) Time-course chemical images of relative Raman intensity of PG-d6 to CA in the film area. The rainbow color in the range of 0.0–4.0 shows the relative intensity calculated with band height ratio of PG-d6 characteristic band to CA characteristic band in CA film region. The depth ranges were cut out with positions of maximum and minimum values in the first derivative of the Raman intensity curve with the CA characteristic band. The scanning film depth is described in nominal focus position. Detailed analytical conditions are described in the Methods section.

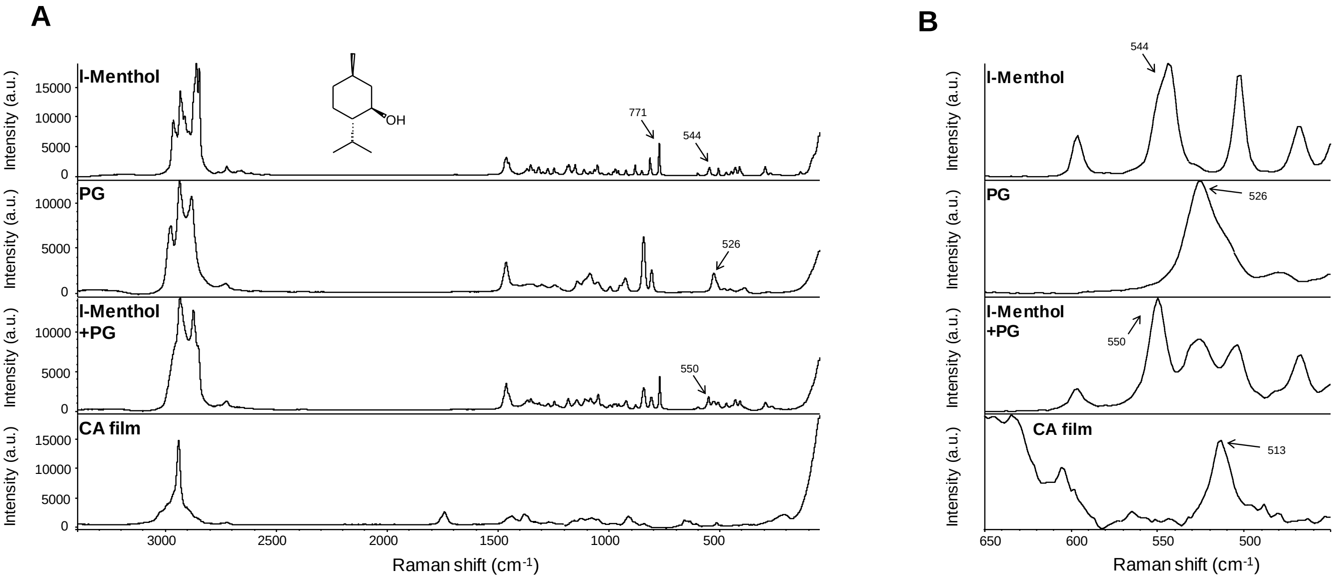


Supplementary Figure S3. (A) Raman spectra of compounds. Raman spectra of l-menthol, PG-d6, l-menthol/PG solution, and CA film acquired using CRM with an exposure time of 1 second at a laser excitation wavelength of 532 nm (power of 10 mW). (B) Enlarged view from wave number range 450 to 650 $cm^{-1}$ in full scale. Detailed analytical conditions are described in the Methods section.

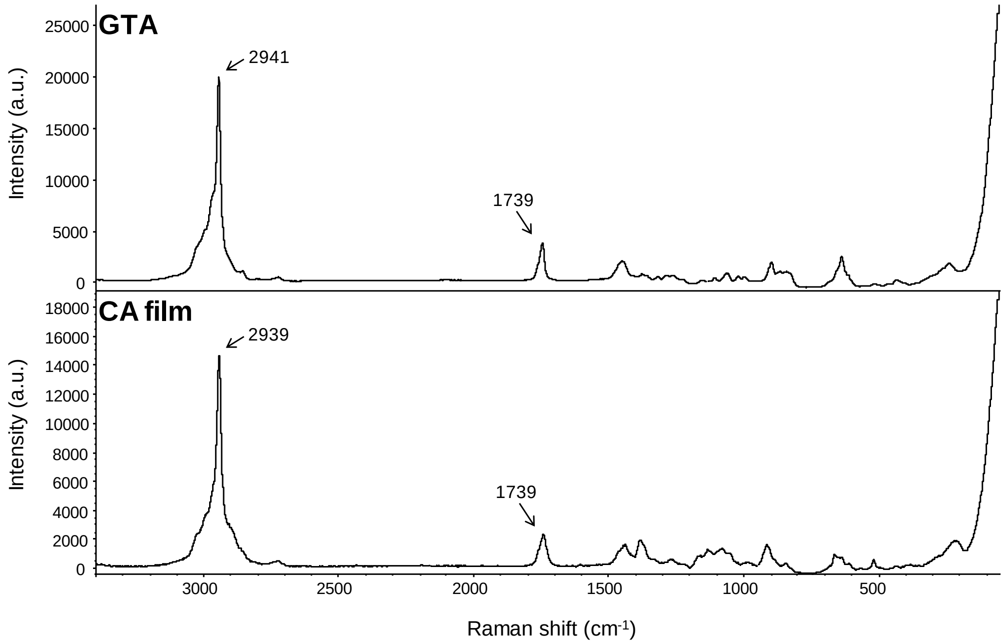


Supplementary Figure S4. Raman spectra of compounds. Raman spectra of GTA and CA film acquired using CRM with an exposure time of 1 second at a laser excitation wavelength of 532 nm (power of 10 mW). Detailed analytical conditions are described in the Methods section.
